# Frequency-Controlled AC-MAO Coatings with Ca, P, and Se on Magnesium: Toward Tailored Surfaces for Biodegradable Implants

**DOI:** 10.3390/ma18245505

**Published:** 2025-12-07

**Authors:** Balbina Makurat-Kasprolewicz, Endzhe Matykina

**Affiliations:** 1 Department of Biomaterials Technology, Gdansk University of Technology, 80-233 Gdańsk, Poland; 2 Departamento de Ingeniería Química y de Materiales, Facultad de Ciencias Químicas, Universidad Complutense, 28040 Madrid, Spain; 3 Unidad Asociada al ICTP-CSIC, Instituto de Química Médica (IQM-CSIC), Grupo de Síntesis Orgánica y Bioevaluación, Instituto Pluridisciplinar (UCM), Paseo de Juan XXIII 1, 28040 Madrid, Spain

**Keywords:** plasma electrolytic oxidation, micro-arc oxidation, magnesium, selenium, coating, biodegradable implants

## Abstract

**Highlights:**

**What are the main findings?**

- AC frequency significantly affected MAO coating morphology and thickness.
- CaPSe_100 coating showed the most uniform structure and highest porosity (28.4%).
- All coatings were strongly hydrophilic (contact angles 8–18°).
- CaPSe_100 achieved a degradation rate of ~0.012 mm/year vs. ~5.50 mm/year for bare Mg.

**What are the implications of the main findings?**

- Frequency tuning enables control of MgO growth and coating uniformity.
- The MAO coating effectively suppresses hydrogen evolution on pure Mg.
- Porous and hydrophilic surfaces may enhance osseointegration potential.
- Findings support the design of durable, multifunctional coatings for Mg implants.

**Abstract:**

The present study investigates the influence of alternating current (AC) frequency on the formation and properties of calcium-, phosphorus-, and selenium-containing micro-arc oxidation (MAO) coatings on high-purity magnesium. Coatings were produced at 50–400 Hz in a phytic-acid-based electrolyte containing Ca, P, and Se precursors, and their structure, chemistry, and functional performance were systematically evaluated. Surface morphology, analyzed by SEM and optical profilometry, revealed frequency-dependent features: lower frequencies (50 Hz) promoted thicker, rougher coatings with extensive cracking, whereas intermediate frequencies (100–200 Hz) yielded more uniform, porous surfaces. The CaPSe_100 specimen exhibited the most homogeneous topography (lowest S10z and SD) combined with the highest porosity (28.4%), strong hydrophilicity, and the greatest selenium incorporation (1.30 wt.%). Hydrogen evolution testing in Hanks’ solution demonstrated a drastic improvement in corrosion resistance following MAO treatment: the degradation rate of bare Mg (5.50 mm/year) was reduced to 0.012 mm/year for the CaPSe_100 coating—well below the clinical tolerance threshold for biodegradable implants. This outstanding performance is attributed to the synergistic effect of a uniform oxide barrier, optimized porosity, and homogeneous surface morphology. The results highlight the potential of frequency-controlled AC-MAO processing as a route to tailor magnesium surfaces for multifunctional, corrosion-resistant biomedical applications.

## 1. Introduction

Biodegradable metallic materials are increasingly regarded as promising candidates for next-generation temporary implants, offering a unique combination of load-bearing capability and gradual resorption in vivo [1]. Among them, magnesium (Mg) and its alloys stand out due to their mechanical properties closely matching those of natural bone, low density, and inherent biodegradability [2]. These features make Mg particularly attractive for orthopedic and cardiovascular applications, where long-term implant persistence is neither required nor desirable [3]. Compared to conventional permanent implant alloys such as stainless steel or titanium, Mg has the additional advantage of eliminating the need for secondary surgery to remove the device, thereby reducing patient risk and healthcare costs [4].

Despite these advantages, the widespread use of Mg-based implants remains limited by their rapid and uncontrolled corrosion in physiological environments. Accelerated degradation can compromise the mechanical stability of the device before tissue healing is complete. In addition, excessive hydrogen evolution (HE) and local alkalization may adversely affect the surrounding tissue response [5]. Therefore, in addition to controlling the degradation rates, it is equally important that Mg-based implants exhibit biofunctional surfaces capable of supporting osseointegration, reducing inflammation, and resisting bacterial colonization [6,7]. Consequently, strategies that can simultaneously modulate the corrosion behavior and enhance the biological performance of Mg are of vital importance for its clinical translation.

Surface modification has emerged as an effective strategy to overcome the inherent limitations of Mg. Among the available approaches, micro-arc oxidation (MAO), also referred to as plasma electrolytic oxidation (PEO), has received considerable attention. This technique enables the formation of hard, adherent, and porous oxide coatings with tunable thickness and composition [8]. In the case of AC-MAO, several processing parameters can be adjusted, among which frequency plays a particularly important role. Frequency strongly influences plasma discharge characteristics, which in turn govern coating morphology, porosity, phase composition, and the extent of element incorporation from the electrolyte [9,10,11]. A further advantage of MAO is its ability to incorporate species from the electrolyte directly into the oxide coating, thereby imparting additional functionality beyond corrosion protection [12].

In the context of biomedical applications, calcium (Ca) and phosphorus (P) are of particular importance, as they constitute the primary inorganic components of natural bone and are directly involved in mineralization processes and osseointegration. Their incorporation into MAO coatings has repeatedly been shown to enhance bioactivity, promote apatite formation, and improve the early interaction of implants with surrounding tissues [13,14]. Moreover, Ca- and P-containing MAO coatings on Mg and its alloys typically exhibit improved corrosion resistance, as the formation of Ca–P-rich amorphous phases can delay electrolyte penetration and enable the controlled release of biologically beneficial ions [15]. Selenium (Se), although less commonly used in micro-arc oxidation systems, has recently gained attention as a promising functional dopant for metallic implants [16,17,18]. Se is an essential trace element involved in antioxidant defense, regulation of cellular redox status, and immune response. Importantly, Se exhibits well-documented antibacterial and anticancer activity at controlled concentrations [17,19], which may help mitigate two major clinical challenges associated with Mg implants: postoperative infection and adverse inflammatory reactions. Incorporating Se into MAO coatings therefore offers the possibility of creating a multifunctional surface that simultaneously enhances corrosion protection, reduces oxidative stress, and provides inherent antimicrobial capacity. Furthermore, the co-incorporation of Ca, P, and Se within a single MAO coating may represent a synergistic strategy [12,16,17,18]: (I) Ca and P support bone regeneration and biological integration; (II) Se provides antimicrobial and antioxidant functions; (III) together they yield a coating that is simultaneously bioactive, protective, and therapeutically functional. Despite these advantages, the combined addition of Ca, P, and Se in AC-MAO systems on Mg remains to be explored. Addressing this gap provides not only scientific novelty but also direct relevance for the rational design of next-generation biodegradable magnesium implants.

In this study, we investigated the influence of alternating current (AC) frequency, a key process parameter in micro-arc oxidation (MAO), on the properties of coatings formed on high-purity magnesium, using a specially designed, bioactive electrolyte enriched with calcium, phosphorus, and selenium. Several studies have examined the role of AC frequency in MAO processing, primarily on aluminum and titanium alloys. For example, Zhu et al. [20] demonstrated that increasing frequency reduced single-discharge energy and improved corrosion resistance of MAO coatings on Al alloys, while Zhao et al. [21] showed that higher frequencies promote denser, more uniform oxide coatings. Similarly, Sobolev et al. [22] reported enhanced compactness and corrosion resistance of MAO coatings on Ti-6Al-4V at elevated frequencies. However, such studies have focused on structural alloys, with virtually no research addressing frequency-controlled incorporation of biofunctional elements, particularly Ca, P, and Se, into MAO coatings on pure magnesium destined to biodegradable implant applications. To address this gap, the present work systematically examines how AC frequency governs the incorporation efficiency of biologically relevant species and influences surface characteristics and functional performance. We evaluated coatings fabricated under varying frequencies (50–400 Hz), focusing on their morphology, thickness, elemental and phase composition, porosity, wettability, and surface uniformity. The most promising coating—CaPSe_100—was selected based on a combination of physicochemical and structural parameters, and subjected to HE testing under simulated physiological conditions. This allowed for direct assessment of its degradation rate and corrosion resistance relative to bare magnesium. By linking the AC-MAO process parameters to key coating characteristics and in vitro degradation behavior, this work aims to support the development of multifunctional, corrosion-resistant, and biologically favorable coatings for future research on magnesium-based implants.

## 2. Materials and Methods

### 2.1. Materials and Reagents

High-purity magnesium (99.99%; Gangya Metal, Jiangsu, Co., Ltd., Wuxi City, China) was cut from a rod with a diameter of 16 mm into cylindrical specimens measuring 9 mm in height. Prior to surface treatment, the samples were ground sequentially with abrasive paper up to #1200 grit, followed by ultrasonic cleaning in 2-propanol for 15 min. Immediately before the PEO process, specimens were rinsed with distilled water. The MAO suspension was prepared from the following commercially available chemicals (Table 1): sodium hydroxide (NaOH) and calcium dihydrogen phosphate monohydrate (Ca(H_2_PO_4_)_2_·H_2_O) from PanReac (Barcelona, Spain); a 50 wt.% aqueous phytic acid (C_6_H_18_O_24_P_6_) solution from Sigma-Aldrich (Saint Louis, MO, USA); and sodium selenite (Na_2_SeO_3_) from Thermo Scientific (Waltham, MA, USA).

### 2.2. Experimental Conditions of the AC-MAO Treatment

The AC micro-arc oxidation (MAO) treatments were conducted using a regulated 2 kW AC power supply (EAC-S2000, ET Systems Electronic, Altlußheim, Germany) connected to a double-walled 2 L glass reactor maintained at 20 °C. A 316 stainless steel plate (7.5 × 15 cm) was employed as the counter electrode. The process was conducted under a square-wave AC regime with an anodic voltage of 350 V and a cathodic voltage of 50 V for 5 min, while the RMS current density was maintained at 0.12 A/cm^2^. The applied voltage was gradually ramped up to its full amplitude within the first 60 s of operation. The experimental variable was the AC MAO frequency, as summarized in Table 1, selected based on established low-frequency MAO conditions [13] and the operational frequency range of the equipment (50–500 Hz). Voltage and current waveforms were continuously recorded using a 16-bit Keithley KUSB-3116 data acquisition system (Keithley Instruments, Inc., Solon, OH, USA) (sampling interval: 0.1 s) controlled via LabView v.8.5 software (National Instruments, Austin, TX, USA). After the MAO treatment, all specimens were rinsed with distilled water and dried in ambient air.

### 2.3. Characterization

To prevent charging effects during SEM observation, all specimens were sputter-coated with a thin carbon layer. Surface morphology was examined using a JEOL JSM-6400 scanning electron microscope (SEM; JEOL Ltd., Tokyo, Japan) operated at 20 kV. Pore size and surface porosity of the MAO coatings were quantified by image analysis of backscattered electron (BSE) SEM micrographs at 1000× magnification with ImageJ v1.5.3t software. The images were converted to binary format and contrast-adjusted to ensure accurate pore segmentation and reliable evaluation of pore distribution.

The elemental composition of the coatings was examined using energy-dispersive X-ray spectroscopy (EDS) (Oxford Link Pentafet 6506, Oxford Instruments, Abingdon, UK) integrated into a JEOL JSM-6400 scanning electron microscope (JEOL Ltd., Tokyo, Japan), operated with a standard detector configuration at an accelerating voltage of 20 kV.

Phase composition was determined by X-ray diffraction (XRD) using an X’Pert PRO MPD diffractometer (Malvern Panalytical, Malvern, UK) equipped with Cu Kα radiation. Diffraction patterns were recorded over the 2θ range of 10–80°, with a step size of 0.033° and a counting time of 100 s per step. The data were processed by background subtraction and peak fitting of the reflections. For quantitative analysis overlapping peaks in the 36–37° 2θ region were deconvoluted using Gaussian functions. Relative phase fractions of Mg and MgO were estimated from the integrated peak areas and expressed as percentages of the total diffracted intensity.

Surface topography (*n* = 3) was characterized with a focus-variation optical profilometer (InfiniteFocusSL, Alicona GmbH, Graz, Austria) equipped with a ×10 objective and controlled via the IFMeasure Suite v5.3 software. Areal roughness and functional surface parameters were derived from these measurements.

Coating thickness (*n* = 10) was determined as the mean of ten independent measurements performed with a Fischer ISOSCOPE-FMP10 eddy-current instrument (ISOSCOPE FMP10, Fischer Instruments, Barcelona, Spain) with an FTA3.3H probe.

Water contact angle (*n* = 3) was measured using an FTA1000 Drop Shape Analysis System of First Ten Angstroms (Newark, CA, USA) equipped with a high-speed Edmund Optics 5582 camera and a Navitar lens, operated via the FTA32 interface. Droplets of ~2 µL were deposited onto the surface, and measurements were performed on three different specimens.

To examine the internal structure of the coatings, the specimens were sectioned, embedded in epoxy resin, and sequentially ground and polished. The cross-sections of all anodized specimens were analyzed using a scanning electron microscope (Tescan Vega G4, Brno, Czech Republic) operated at 5 kV in backscattered electron (BSE) mode. In addition, cross-sections of the specimens after corrosion assays (uncoated Mg and CaPSe_100) were prepared following the same procedure to evaluate coating integrity and potential degradation after immersion.

HE (*n* = 3) was monitored for both bulk magnesium and PEO-coated specimens over a 14-day immersion period in Hanks’ balanced salt solution (HBSS) prepared according to the composition reported in [23]. The specimens were positioned in 25 (modified specimens)/50 mL (uncoated specimens) burettes placed inside a 12 L plastic chamber connected to an 11 L reservoir. The pH of the physiological solution was maintained at 7.4 by automatic regulation with a CO_2_ flow controlled through a pH sensor and switch system. Selected specimens were tested in triplicate. The total exposed surface area of the specimens was ~8.4 cm^2^. A schematic representation of the experimental setup is available in [24]. Furthermore, degradation rates were calculated using the HE method. Degradation rate (DR) was calculated following the approach commonly used in Mg corrosion studies [25,26], which is also in line with ISO 10993-15 recommendations [27] for evaluating degradation products of resorbable metals:

$${DR}_{H_{2}}=\frac{2.279\cdot V_{H_{2}}}{A\cdot t}$$

where $V_{H_{2}}$ is the collected volume of hydrogen (mL), A is the exposed surface area (8.4 cm^2^), and t is the immersion time (14 days).

### 2.4. Statistical Analysis

Statistical analyses were performed using SigmaPlot 15.0 (Systat Software, Chicago, IL, USA). Data were assessed for normality using the Shapiro–Wilk test implemented in the software. For datasets meeting parametric assumptions, one-way ANOVA followed by Bonferroni’s *t*-test was applied for multiple comparisons. Results are expressed as mean ± standard deviation (SD), with statistical significance set at *p* < 0.05.

## 3. Results and Discussion

### 3.1. Voltage–Current Behavior in AC-MAO Coatings Treatments

**Figure 1:**
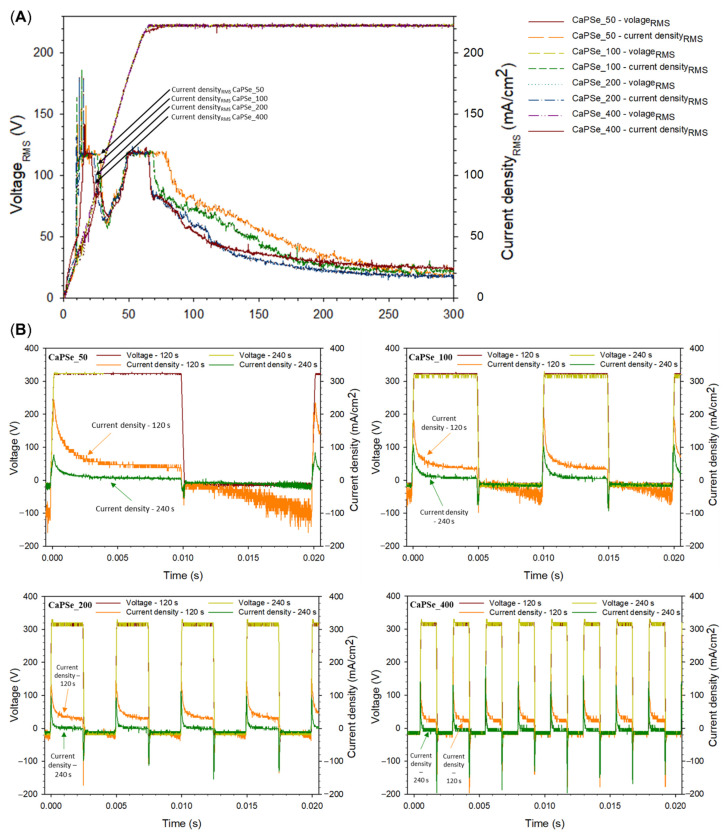
(**A**) Root mean square (RMS) voltage and current density as a function of time during the MAO processes. (**B**) Instantaneous voltage and current density waveforms recorded by oscilloscope at 120 and 240 s of the MAO treatment.

Figure 1A illustrates the evolution of RMS voltage during the PEO treatments. A characteristic inflection point corresponds to the end of the applied ramp, after which the voltage gradually stabilized at ~220 V_RMS_. This value is slightly lower than the theoretical prediction (V_RMS_ = $\frac{\sqrt{350^{2}+50^{2})}}{2}$ = 250 V), which can be attributed to the progressive increase in coating impedance during the process [28]. It should be noted that direct visual observation of plasma discharges was not feasible in this study, as the process was conducted in a suspension rather than a transparent electrolyte. Interestingly, for all frequencies, the current density exhibited a transient decrease to ~60 mA·cm^−2^ within the first 20–50 s of treatment. The duration of this drop increased with frequency, possibly reflecting delayed stabilization of discharge activity at higher switching rates. Such early-stage current decay could be linked to charging effects at the coating–electrolyte interface and to the formation of an initial dielectric barrier layer, which temporarily suppresses discharge activity [29,30]. Moreover, the apparent energy delivered during the MAO process was quantified based on the rms voltage–time and rms current–time transients (Figure 1A), as well as the real instantaneous energy calculated over a 20 ms window at the 120 s time point (Figure 1B). For each sampling interval, the incremental energy input was calculated as E_i_ = |V_i_⋅I_i_|⋅Δt_i_, where Δt_i_ is the time difference between consecutive data points. The total energy was obtained by summing all increments over the entire treatment duration and normalizing by the specimen surface area. Based on this analysis, the apparent specific energy inputs followed the sequence: ~1.17 kWh/(m^2^μm) (CaPSe_400) > ~1.09 kWh/(m^2^μm) (CaPSe_50) > ~1.07 kWh/(m^2^μm) (CaPSe_100) > ~0.93 kWh/(m^2^μm) (CaPSe_200). Remarkably, these values are 3–8 times lower than those reported by Moreno at al. [31] for AC PEO of h.p. Mg in a suspension electrolyte. This means that the microdischarge events in the present work are more energy efficient; i.e., more oxide material is generated per unit of electrical charge. Further, at the 120 s time point, when the decaying current was still sustaining the microdischarge events (as evident by the characteristic acoustic noise), the real instantaneous energy over 20 ms showed the following order: ~6.5 Wh/m^2^ (CaPSe_100, CaPSe_200) > ~6.2 Wh/m^2^ (CaPSe_400) > ~4.5 Wh/m^2^ (CaPSe_50). This behavior is consistent with the highly nonlinear and self-regulating nature of the MAO process [12]. In MAO, the electrical energy supplied to the surface is not converted uniformly into oxide growth; instead, it is partitioned among plasma heating, dielectric breakdown events, gas evolution, charge accumulation, and dynamic changes in interfacial impedance [32,33]. As the coating thickens, its electrical resistance increases, altering the intensity, duration, and spatial distribution of microdischarges. Consequently, a higher apparent energy input—such as that observed for CaPSe_400—does not necessarily produce a thicker or more porous coating. This further supports the idea that oxide-growth efficiency is governed primarily by the temporal behavior of microdischarges and the evolving dielectric properties of the growing layer, rather than by the total energy supplied to the system.

Analysis of instantaneous voltage and current waveforms provided additional insights into the nonlinear electrical response of the system (Figure 1B). While no major differences were observed between the frequency conditions, subtle trends were identified: (i) CaPSe_100 and CaPSe_400 exhibited the highest anodic peak current densities at ~300 mA/cm^2^ after 120 s, compared to ~220 mA/cm^2^ for the other coatings; (ii) CaPSe_50 displayed the lowest anodic peak current density at ~80 mA/cm^−2^ after 240 s; and (iii) CaPSe_400 showed the highest cathodic peak currents, reaching ~150–200 mA/cm^2^ at 120 s and ~140–180 mA/cm^2^ at 240 s. Elevated peak currents are generally indicative of more intense microdischarge activity, which may correlate with increased local melting and element incorporation [28]. The observed variations in current transients suggest that frequency-dependent discharge behavior is governed more strongly by charge accumulation and relaxation dynamics at the coating/electrolyte interface than by bulk electrolyte properties [34]. To fully elucidate these mechanisms, further studies employing high-speed imaging of discharge events) in combination with optical emission spectroscopy using transparent electrolytes are required. Such approaches would provide valuable information on the influence of frequency on plasma characteristics, ion transport, and the capacitive response of the growing coating.

### 3.2. Morphology and Chemical Composition

**Figure 2:**
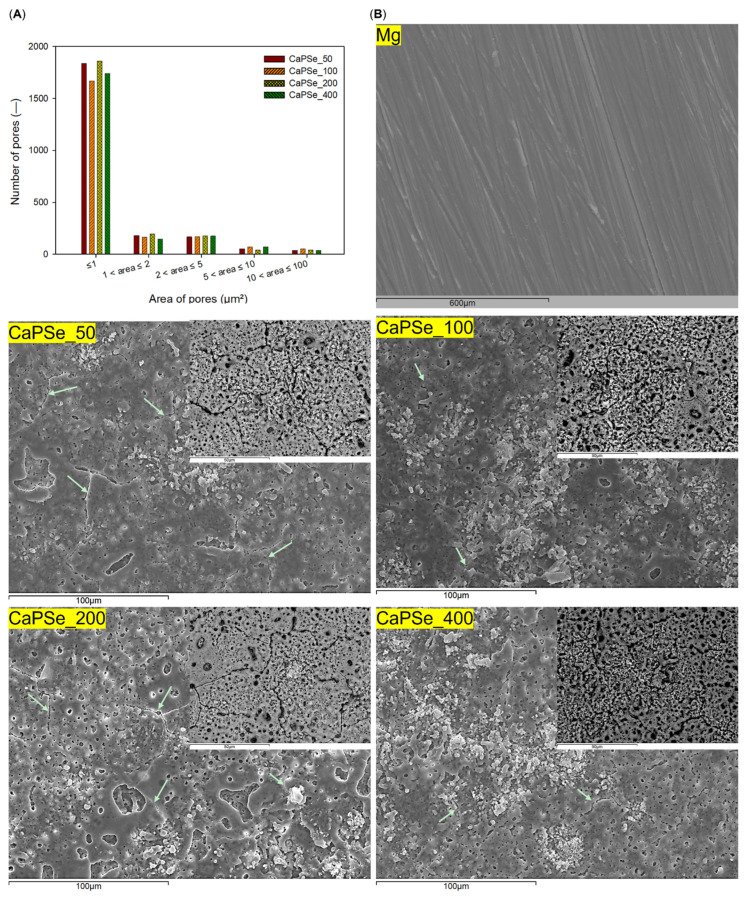
(**A**) Pore-size distribution of the MAO-modified specimens, quantified using ImageJ software from backscattered electron SEM micrographs acquired at 1000× magnification. (**B**) SEM micrographs of uncoated Mg and MAO-coated substrate. Large images were acquired in secondary electron mode at 1000× magnification. For the modified specimens, additional backscattered electron micrographs at 3000× magnification are shown in the upper right corner. Green arrows indicate cracks in MAO coatings.

Figure 2 presents SEM micrographs of the surface morphology of uncoated Mg and MAO-coated specimens. In contrast to the relatively smooth surface of bare magnesium, all modified samples exhibited a markedly more developed structure. As is typical for MAO, the coatings contained microcracks, most likely formed during rapid solidification and contraction of the oxide [14]. Notably, among all coatings, CaPSe_100 displayed the most favorable morphology: it showed the fewest cracks, indicating a more mechanically stable surface, whereas CaPSe_50 and CaPSe_200 exhibited wide, interconnected cracks that may compromise coating integrity. In several regions of these latter coatings, a layered structure with underlying porosity was visible beneath the cracks, suggesting locally intensified discharges during the MAO process. All MAO coatings exhibited the typical dual-scale pore architecture—coexisting small and large pores—together with a homogeneous distribution of precipitates. In BSE mode, these precipitates appeared as bright, white-toned features, consistent with selenium enrichment, as selenium produces strong atomic-number contrast in backscattered electron imaging. Although the qualitative appearance of pore morphology was similar across all specimens, the minor numerical differences observed in the quantified pore-area distributions likely reflect subtle local variations within the MAO coatings. The average pore area (Table 2) ranged from ~1.00 μm^2^ (CaPSe_200) to ~1.32 μm^2^ (CaPSe_100). Importantly, CaPSe_100 combined the largest mean pore size with the highest porosity level (28.4%), a combination highly beneficial for biomedical applications. While most pores across all coatings were smaller than 1 μm^2^—a feature that may hinder bacterial adhesion, given that pathogens such as *Escherichia coli* [35] and *Staphylococcus aureus* (~1 μm) [36] are of comparable size—the higher overall porosity of CaPSe_100 is expected to enhance host–material interactions. Although osteoblasts are larger (20–50 μm) [37], initial attachment occurs via nanoscale contact and filopodia, which extend into surface irregularities to establish adhesion [38]. Micropores smaller than 10 μm are particularly advantageous, as they increase the available surface area, promote protein adsorption, and stimulate osteogenic differentiation, thereby creating a favorable environment for bone tissue regeneration [39]. Together, these findings suggest that the limited crack density, optimal pore architecture, high porosity, and selenium-rich precipitates of the CaPSe_100 coatings can provide a dual benefit: limiting bacterial colonization while simultaneously enhancing bioactivity.

**Figure 3:**
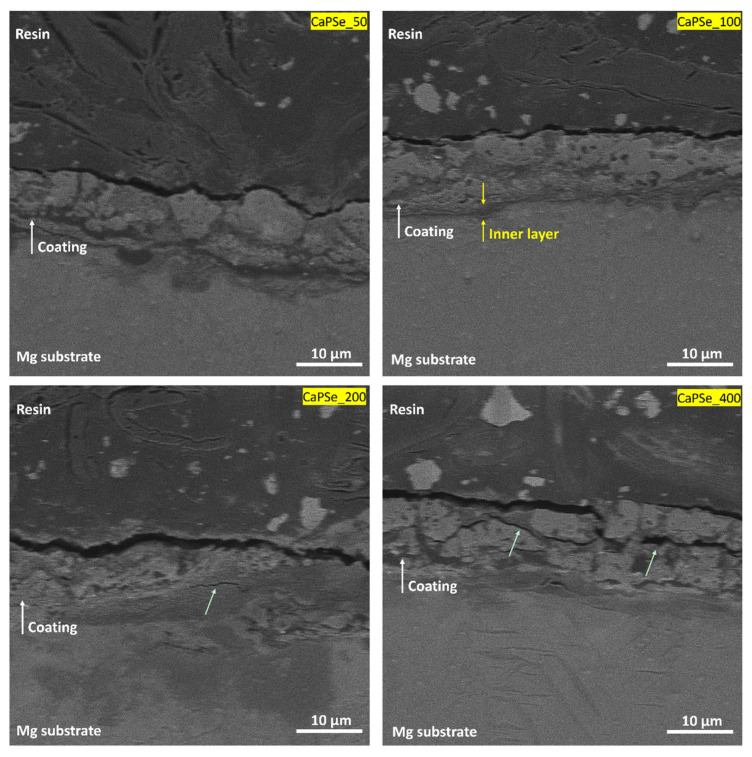
Cross-sectional SEM images (at 5000× magnification) of the MAO coatings. Green arrows highlight cracks and interlayer defects visible in selected coatings. The CaPSe_100 specimen shows the most uniform and compact structure.

Cross-sectional observations (Figure 3) revealed that all MAO coatings exhibited a typical porous structure firmly adhered to the magnesium substrate. Among all the specimens, the CaPSe_100 coating appeared the most uniform in terms of thickness, showing no significant penetration of pores into the underlying substrate and no upward bulging of the oxide layer. This morphological homogeneity suggests a stable growth process. In contrast, the coatings produced at higher frequencies, particularly CaPSe_200 and CaPSe_400, displayed a greater number of structural defects such as horizontal cracks and interlayer discontinuities. These features are commonly attributed to internal stress accumulation and rapid solidification during plasma discharge events [14], and they can compromise the long-term barrier integrity by facilitating localized electrolyte ingress. Furthermore, for CaPSe_100, a clearly distinguishable inner layer was visible beneath the outer porous region. This compact zone likely represents the dense barrier layer typically formed during MAO, which plays a critical role in corrosion protection by limiting ion transport between the coating and substrate [15]. In contrast, for the other coatings, identifying a well-defined inner layer was more challenging, as no distinct transition region was observed between the porous and dense zones.

**Figure 4:**
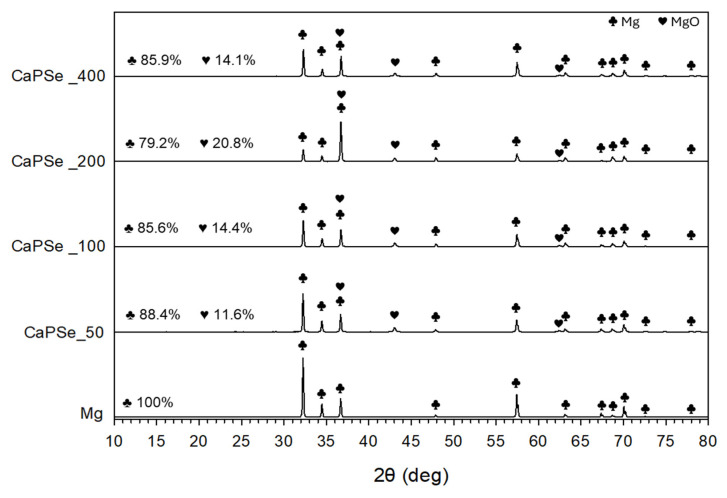
XRD spectra of uncoated and MAO-coated magnesium specimens. All modified specimens contained reflections from metallic Mg originating from the substrate.

EDS analysis (Table 3) confirmed the incorporation of Ca, P, Na, and Se from the suspension. Phosphorus was abundant in all coatings (13.10–13.78 wt.% for CaPSe_100 and CaPSe_200), while calcium content peaked in CaPSe_50 and was lowest in CaPSe_400. This trend is consistent with the longer cathodic half-cycles at low frequency, which favor Ca^2+^ migration and uptake [40,41]. Sodium was detected at 3.33–4.07 wt.%, reflecting both its intentional addition to influence morphology and its easy incorporation during the cathodic phase. Selenium was also incorporated into the coatings, reaching a maximum of 1.30 wt.% in CaPSe_100, albeit at lower levels than P and Na. Previous study [16] indicates that Se exhibits a distinct dose-dependent biological response: high incorporation levels in MAO coatings (~14 wt.%) induced cytotoxicity, whereas moderate levels (~8 wt.%) promoted osteogenic differentiation and antibacterial activity. The Se content obtained in this work lies well within the biologically safe range reported in the literature and is therefore not expected to pose cytotoxic risk. Notably, the limited Se uptake observed here is consistent with the lower solubility of SeO_3_^2−^ species and their competitive incorporation during the anodic stage. Importantly, when normalized to sodium incorporation, CaPSe_100 exhibited the highest Se/Na ratio, clearly indicating that under these conditions selenium uptake was most efficient relative to sodium. This is particularly relevant from a biomedical perspective, since even small amounts of selenium are known to provide strong antioxidant, antibacterial, and potentially anticancer functionality [16,42,43]. The uptake mechanisms of the different electrolyte species can be rationalized by their ionic charge: Ca^2+^ and Na^+^ are incorporated predominantly during the cathodic half-cycle, whereas PO_4_^3−^ and SeO_3_^2−^ migrate toward the substrate during the anodic half-cycle. Consequently, Ca was most effectively incorporated at 50 Hz, while selenium enrichment peaked at 100 Hz—precisely the condition corresponding to the CaPSe_100 coating. The Ca/P ratio (0.08–0.11) obtained for all coatings is typical for MAO coatings on magnesium [14] and falls well below the stoichiometric value of 1.67 for crystalline hydroxyapatite. This deviation suggests the formation of non-stoichiometric, likely amorphous calcium phosphate phases. Such amorphous structures are known to be more soluble and capable of releasing Ca^2+^ and PO_4_^3−^ ions, thereby enhancing biological activity and promoting mineralization [44]. The absence of crystalline Ca–P peaks in the XRD patterns (Figure 4) supports this interpretation. Further, the Ca/Mg (0.03–0.04) and P/Mg (0.37–0.39) ratios were comparable across all coatings, indicating consistent incorporation relative to the metallic substrate.

### 3.3. Phase Composition

In all specimens, metallic Mg originating from the substrate was detected as the dominant phase (Figure 4). Quantitative XRD analysis further revealed that the relative proportion of MgO increased with increasing AC frequency, reaching its maximum at 200 Hz (~21%), whereas at 50 Hz, 100 Hz, and 400 Hz the MgO content remained lower (~12–14%). This indicates that discharge characteristics at intermediate frequencies promote more extensive oxidation of the Mg substrate. The presence of MgO is generally beneficial, as it forms a protective barrier that slows down degradation and reduces HE. Nevertheless, excessive oxidation and the formation of a thick, brittle oxide layer can promote crack initiation and growth; once the barrier is disrupted, corrosive media penetrate through pores and cracks, accelerating localized corrosion of the underlying Mg substrate [45,46,47]. Crystalline phases containing Ca, P, or Se were not observed. Their absence may be explained by several factors, including the relatively low concentration of these elements in the coatings, their incorporation in amorphous or nanocrystalline states, or their presence in amounts below the detection limit of laboratory XRD [48,49]. It should be noted that reflections from calcium phosphates or selenium species would typically appear in the 2θ range of ~23–35° [50,51,52]; however, no distinct peaks were resolved in this region, suggesting that the incorporated species are either poorly crystalline or dispersed within the oxide matrix. This interpretation is consistent with earlier publications [53,54], where surface-sensitive techniques such as EDS, XPS, or FTIR were required to confirm the presence of bioactive dopants not visible by XRD. Such a chemical state can be advantageous, as amorphous phases are often more bioactive and resorbable than their crystalline counterparts, potentially enhancing osseointegration [44,55].

### 3.4. Thickness and Topography

The coating thickness (Table 2) decreased systematically with increasing AC frequency, a trend commonly attributed to the reduced duration and energy of individual microdischarge events at higher switching rates. At lower frequencies, longer anodic and cathodic half-cycles permit greater charge accumulation and higher discharge energy, promoting deeper dielectric breakdown, more extensive localized melting, and consequently thicker oxide growth. In contrast, as the frequency increases, pulse durations become too short to sustain high-energy discharges, resulting in lower plasma temperatures and a thinner, more compact coating. In the present study, the maximum coating thickness was achieved for CaPSe_50 (~8.6 µm), whereas the thinnest coating formed at 400 Hz (~6.5 µm). Notably, these values are substantially higher than those reported by Shi et al. [17], who employed a high-frequency MAO process (2000 Hz) on WE43 magnesium alloy and obtained coatings of only 2.68 µm (One-MAO) and 3.21 µm (Two-MAO). This comparison underscores the strong influence of frequency on coating growth efficiency and highlights the capability of low-frequency AC-MAO to generate significantly thicker bioactive coatings.

**Figure 5:**
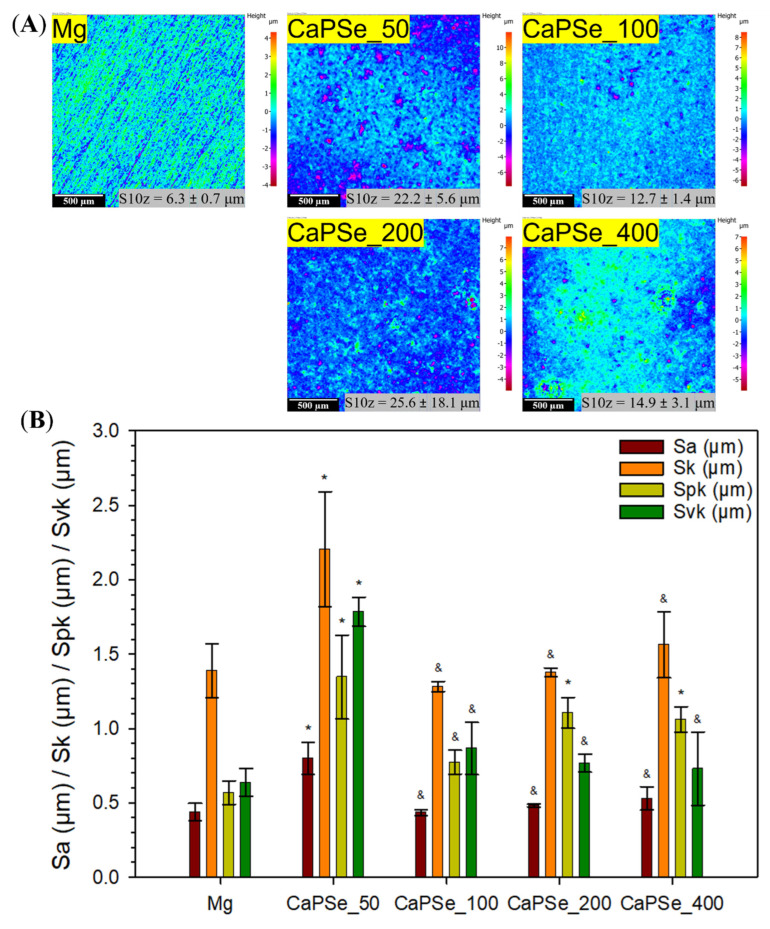
(**A**) Topographical maps of all specimens with ten-point height (S10z) values. (**B**) Selected topographical parameters (*n* = 3) obtained by optical profilometry for uncoated Mg and MAO-coated Mg. * Statistically significant difference compared to uncoated Mg (*p* < 0.05); ^&^ statistically significant difference compared to CaPSe_50 (*p* < 0.05). No other statistically significant differences were found between groups.

Figure 5A presents 2D surface topography maps of the specimens before and after the MAO process. For the uncoated magnesium substrate, characteristic grinding marks are clearly visible. In contrast, the MAO-modified samples exhibit more complex morphologies with pronounced irregularities, confirming the formation of porous coatings, which is typical for the MAO process [14,50,56]. Such irregularities have been reported to act as preferential sites for discharge events during coating growth, as noted by Nashrah et al. [57]. The highest surface roughness (Sa) was recorded for CaPSe_50, which can be explained by the longer cathodic half-cycle at 50 Hz, enhancing the migration and incorporation of Ca^2+^ ions [40,41]. At higher frequencies, shorter cathodic pulses likely limited this process, resulting in Sa values of CaPSe_100, CaPSe_200, and CaPSe_400 comparable to the unmodified substrate (Figure 5B). Since Sa provides only a general descriptor of surface topography, the S10z parameter was also evaluated. The highest S10z (25.6 μm) was observed for CaPSe_200, but the very large standard deviation (~18 μm) indicated significant heterogeneity and uneven deposition. By contrast, CaPSe_100 showed the lowest S10z value and the smallest standard deviation (12.7 ± 1.4 μm), clearly reflecting the most uniform and homogeneous coating among all tested conditions. This uniformity is particularly advantageous, from a corrosion standpoint, as low peak-to-valley variation reduces local stress concentrations and minimizes weak points where electrolyte can penetrate and initiate localized breakdown [58].

Additional parameters with direct tribological and biomedical relevance were also analyzed; namely, core height (Sk), reduced peak height (Spk), and reduced valley depth (Svk):

- Sk describes the load-bearing core height of the surface, corresponding to the load-bearing portion that distributes stresses once the highest asperities have been removed [58,59].
- Spk reflects the height of asperities likely to be removed during the initial “running-in” stage of contact [60,61].
- Svk corresponds to the depth of valleys, which in tribological systems can serve as lubricant reservoirs; in biomedical applications, such valleys may similarly act as micro-reservoirs for body fluids, proteins, or even bioactive molecules [58,62,63].

CaPSe_50 exhibited significantly higher values for all three parameters compared to uncoated Mg, whereas the other coatings showed results closer to the reference, with only Spk differing statistically. This suggests that modification at 50 Hz produced the most pronounced but also the least controlled surface features. To evaluate coating durability, the Spk/thickness (d) ratio was considered, representing the fraction of the coating susceptible to removal during initial wear [53]. The values followed the order: CaPSe_100 (~10%) < CaPSe_200 (~15%) < CaPSe_50 (~16%) < CaPSe_400 (~17%). Crucially, the low Spk/d ratio of CaPSe_100 indicates that only a small fraction of the coating is mechanically vulnerable, which aligns with its exceptionally low corrosion rate and high stability in Hanks’ solution (see Section 3.6). Interestingly, in our recent MAO studies on commercially pure titanium [58] carried out in an electrolyte containing calcium acetate hydrate and β-glycerophosphate disodium salt pentahydrate, the Spk/d values exceeded 17%. Such a reduced Spk/d ratio is particularly advantageous for biodegradable Mg implants, as minimizing the proportion of easily removable surface asperities directly lowers the likelihood of early coating damage, thereby delaying localized corrosion initiation and extending functional implant lifespan. From a biological perspective, the moderate micro-roughness of CaPSe_100, together with its submicron porosity and high hydrophilicity, creates a surface texture that favors early osteoblast adhesion while minimizing features that could retain bacteria or trigger inflammation [12]. Thus, CaPSe_100 not only exhibited the most uniform surface but also the lowest relative fraction of material prone to early removal, underscoring its superior durability, combined with its structural homogeneity, controlled surface topography, and reduced susceptibility to premature degradation.

### 3.5. Wettability

**Figure 6:**
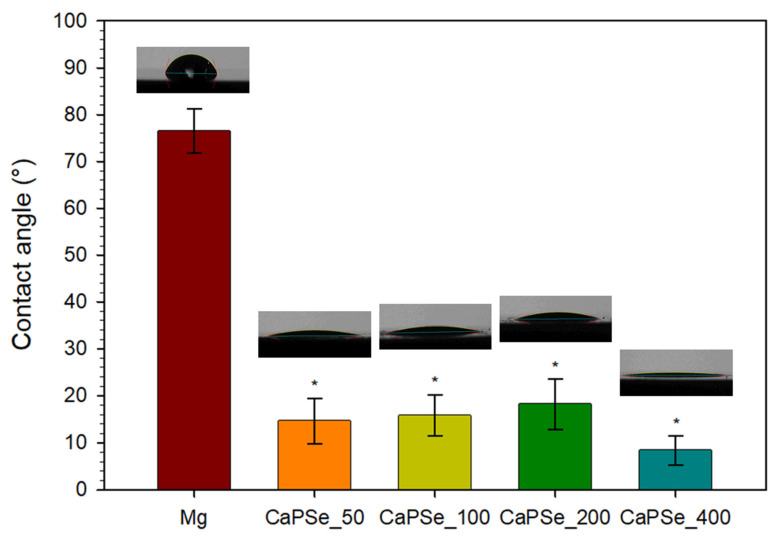
Average contact angle (*n* = 3) for uncoated Mg and MAO-coated Mg with representative images. All specimens were hydrophilic, and surface modification resulted in a significant decrease in contact angle compared to the control. * Statistically significant difference compared to uncoated Mg (*p* < 0.05); no statistically significant differences were observed between coated groups.

ASTM D7334–08 [64] states that a surface is considered hydrophilic when the water contact angle is below 45° and hydrophobic when it exceeds 90°. All MAO-modified specimens in this study (Figure 6), therefore, fall into the hydrophilic range. This observation is particularly relevant since the uncoated magnesium exhibited a significantly higher contact angle of ~77°, which may directly affect its biological response. Enhanced hydrophilicity of MAO-coated Mg is highly desirable, as it has been associated with improved protein adsorption, accelerated osteoblast adhesion, and promotion of osseointegration [65,66]. In contrast, hydrophobic surfaces generally provide more favorable conditions for bacterial adhesion, suggesting that the hydrophilicity of the coatings may also help reduce infection risk [12,67,68]. Wettability is governed by multiple factors, including chemical composition, surface topography, pore distribution, and pore size [66,69], making it difficult to establish a simple correlation. In this study, the water contact angle decreased drastically after MAO modification, confirming a clear transition from ~77° to strongly hydrophilic behavior. The observed differences in water contact angle (8–18°) between the MAO coatings were small, indicating that all surfaces were strongly hydrophilic. Given that all coatings exhibited similarly high open porosity (23–28%), the rapid spreading of the droplet is most plausibly governed by liquid uptake into the interconnected pore network, which increases the effective solid–liquid contact area and accelerates droplet flattening. This interpretation is consistent with previous reports [12,70] on highly porous MAO coatings, where roughness- and porosity-driven wetting has been linked to very low apparent contact angles. The overall high hydrophilicity of all coatings is beneficial from a biomedical perspective, as it facilitates protein adsorption and initial cell adhesion on magnesium-based substrates [12].

### 3.6. Hydrogen Evolution

HE is one of the most physiologically relevant methods for assessing the degradation of magnesium-based biomaterials, as it directly reflects the electrochemical corrosion pathway of Mg (Mg + 2H_2_O → Mg(OH)_2_ + H_2_) [71]. Excessive hydrogen release remains a major limitation for the clinical translation of biodegradable Mg implants, as the formation of gas cavities around the implant site can impair tissue healing and osseointegration [5]. In this study, the CaPSe_100 specimen was selected as the clinically oriented candidate for HE testing due to its favorable combination of structural, chemical, and functional attributes. It exhibited the most uniform surface topography (lowest S10z and SD) along with the highest porosity (28.4%)—a combination advantageous for both controlled degradation and enhanced cell attachment. Chemically, it demonstrated the highest selenium incorporation, indicating efficient anionic uptake despite competition from phosphate and hydroxide species. This is particularly relevant, as selenium provides multifunctional benefits, including antioxidant defense, osteogenic stimulation, and antibacterial activity [16,17,18]. Its intermediate MgO content further indicated a protective barrier effect, without the excessive brittleness and heterogeneity observed at 200 Hz. Collectively, these features provided a rational basis for selecting CaPSe_100 for direct comparison with bare Mg.

**Figure 7:**
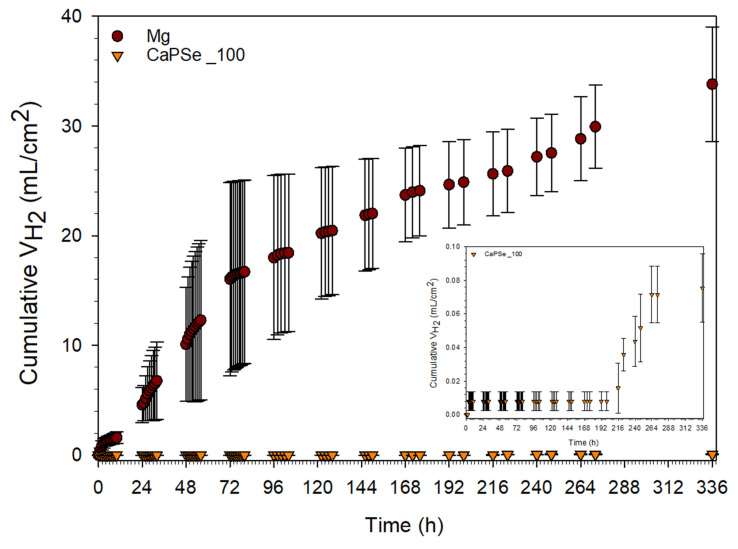
Hydrogen volume (*n* = 3) evolved from uncoated Mg and MAO-coated specimens during 14 days of immersion in Hanks’ solution at 37 °C, with an inset showing a magnified view of the CaPSe_100 specimen.

As shown in Figure 7, the application of AC-MAO treatment led to a substantial suppression of HE compared to bare magnesium. Based on HE measurements, the degradation rate of uncoated Mg during two weeks of immersion is equivalent to approximately 5.504 ± 0.851 mm/year, which is consistent with literature data for pure magnesium in chloride-containing physiological environments [24,72]. Such a rapid degradation rate confirms the well-known limitation of unprotected Mg—its tendency toward uncontrolled corrosion, leading to excessive hydrogen release and local alkalization that can compromise tissue healing. Importantly, the relatively large standard deviations observed for uncoated Mg arise from the intrinsic variability of early-stage hydrogen evolution. Small differences in initial pit activation and surface reactivity lead to markedly different initial corrosion rates, which later converge during prolonged immersion. In stark contrast, the CaPSe_100-coated specimen exhibited an exceptionally low degradation rate equivalent to 0.012 ± 0.003 mm/year during the second week of immersion, corresponding to a reduction of more than three orders of magnitude. Remarkably, the onset of corrosion was not observed during the first 8 days. This striking improvement highlights the strong protective performance of the AC-MAO coating formed at 100 Hz. For reference, MAO coatings typically reduce hydrogen-evolution-derived corrosion rates to the 10^−1^ mm/year range [15]; therefore, the obtained rate in the 10^−2^ mm/year range places CaPSe_100 well below the conventional performance envelope. Moreover, this value lies far beneath the clinical tolerance threshold of ~0.023 mm/year, equivalent to 0.01 mL/cm^2^·day of hydrogen release, generally accepted as safe for biodegradable Mg implants [5]. This result for the MAO modification is highly encouraging. The suppression of HE mitigates two of the most clinically critical complications associated with Mg degradation at the early stages of implantation— gas-pocket formation and local alkalization, both of which can impair tissue healing and hinder implant integration [73]. However, it should be emphasized that HE measurements primarily quantify the extent of degradation, rather than revealing the mechanistic pathway by which corrosion proceeds. Although HE testing is widely regarded as one of the most physiologically relevant methods for evaluating biodegradable Mg—due to its direct reflection of the Mg corrosion reaction—it does not resolve contributions from barrier-layer breakdown, localized microgalvanic effects, or charge-transfer phenomena at the coating–electrolyte interface [74]. For this reason, future work should include complementary electrochemical characterization. Techniques such as potentiodynamic polarization (PDP) and electrochemical impedance spectroscopy (EIS) would provide deeper insight into the corrosion kinetics and the evolution of the inner barrier layer. Importantly, such tests should be performed not only in simple electrolytes but also in more complex, physiologically relevant media (e.g., DMEM containing drugs and proteins), where adsorption processes and protein-layer formation may strongly influence both coating stability and ion transport.

**Figure 8:**
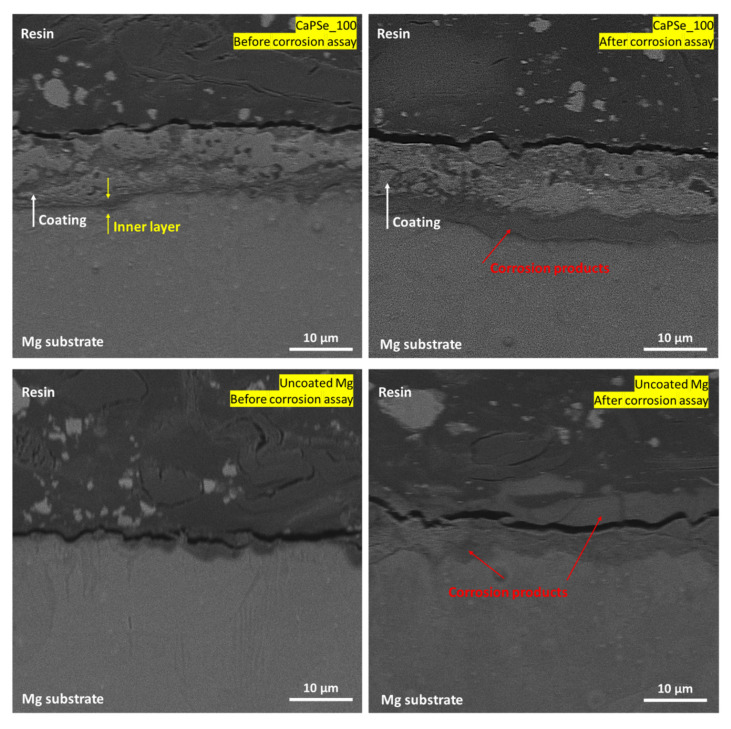
Cross-sectional SEM images (5000× magnification) of uncoated Mg and MAO-coated (with region showing the most pronounced signs of corrosion) specimens before and after 14 days of immersion in Hanks’ solution at 37 °C. The MAO coating remained adherent and structurally intact after immersion.

Cross-sectional SEM analysis performed after 336 h of immersion (Figure 8) revealed the presence of a hydration zone extending parallel to the coating–substrate interface, observed for both uncoated Mg and the CaPSe_100 specimen. This hydrated region corresponds to the formation of corrosion products, mainly Mg(OH)_2_, at the early stage of corrosion, yet its high compactness and uniform distribution beneath the coating, rather than localized attack, indicates that degradation proceeded in a controlled manner without coating delamination. Conversely, in a bare substrate, the corrosion product layer is thick, loose and cracked. It is generally accepted that MAO coatings are inherently porous, consisting of a highly porous outer layer and a thinner, more compact inner layer with nanoscale defects. The latter serves as an effective diffusion barrier, delaying electrolyte penetration to the metallic substrate by significantly reducing ionic transport through the coating and slowing the ingress of aggressive species such as Cl^−^ [15]. This barrier effect limits the formation of underlayer corrosion products and prolongs coating stability during immersion. Consequently, electrolyte access to the substrate typically occurs only through isolated weak points or microdefects formed during individual discharge events. Notably, among all coatings examined in this study, CaPSe_100 exhibited the most continuous and well-defined inner barrier layer, with minimal vertical irregularities and no large undercut regions. This structural integrity likely contributed to its exceptional corrosion suppression during long-term immersion, as the dense inner layer effectively restricted diffusion pathways and delayed the onset of substrate dissolution and hydrogen evolution. As shown in Figure 8, the inner layer of CaPSe_100 remained clearly distinguishable and structurally intact after immersion, further supporting the conclusion that this coating retained both its protective function and mechanical stability throughout the test. The corrosion products layer formation was observed only in limited regions of the metal/coating interface; i.e., in 2 weeks of immersion, an extensive underfilm corrosion has not yet developed. Furthermore, the CaPSe_100 coating integrates several synergistic features—a dense MgO-based barrier, favorable and interconnected porosity, high hydrophilicity, and controlled selenium incorporation—all contributing to its multifunctional performance. These results demonstrate that optimized AC-MAO processing not only enhances the chemical and structural stability of the coating but also ensures controlled, uniform degradation, critical for the safe clinical implementation of magnesium-based implants.

#### Proposed Corrosion Mechanism

**Figure 9:**
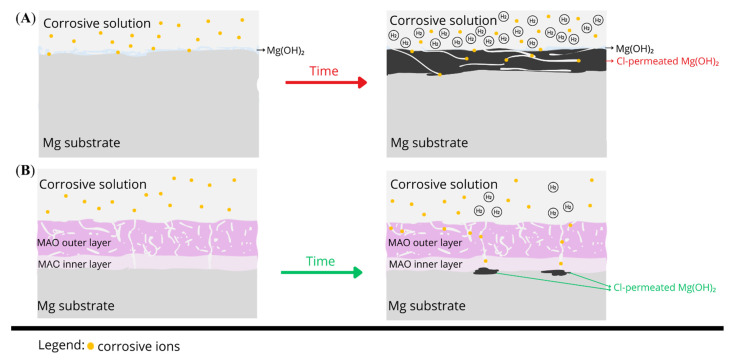
Schematic representation of the proposed corrosion mechanism after 14 days of immersion in Hanks’ solution at 37 °C for: (**A**) uncoated magnesium, showing rapid dissolution, thick and cracked Mg(OH)_2_ corrosion layer formation, and extensive hydrogen evolution; (**B**) MAO-coated magnesium (CaPSe_100), illustrating electrolyte ingress through outer-layer pores, localized under-coating corrosion restricted to isolated barrier-layer defects, and the overall integrity and adhesion of the MAO coating.

The corrosion behavior of uncoated magnesium and AC-MAO-modified magnesium follows two fundamentally different pathways. These mechanisms are illustrated in Figure 9 and consistent with the morphological observations, cross-sectional analyses, and hydrogen-evolution data obtained in this study. Upon immersion in chloride-containing physiological solution, bare Mg undergoes rapid anodic dissolution accompanied by cathodic hydrogen evolution. This reaction sequence produces a thick Mg(OH)_2_ corrosion layer that forms quickly due to local alkalization; however, this layer is highly cracked, poorly adherent, and unable to protect the substrate. Simultaneously, chloride ions penetrate the corrosion layer, creating aggressive microenvironment within the Mg(OH)_2_ which destabilizes the surface [46]. Hydrogen generated beneath the loosened corrosion layer further promotes delamination, leading to a positive feedback loop of accelerated dissolution, extensive corrosion-product build-up, and sustained high hydrogen evolution. This mechanism is reflected in the cross-sections (Figure 8), where uncoated Mg exhibits a thick, fractured corrosion layer and in the hydrogen-evolution results (Figure 7), which indicate rapid and continuous degradation.

In contrast, AC-MAO coatings fundamentally alter this degradation pathway through the presence of a characteristic dual-layer structure consisting of a porous outer layer and a thin but dense inner barrier layer. During early immersion, electrolyte infiltrates the porous outer region primarily through pre-existing discharge-generated microdefects, but the compact inner layer significantly restricts ionic transport. Electrolyte access to the metallic substrate therefore occurs only at isolated weak points where the barrier layer is locally disrupted. In these limited regions, a thin Mg(OH)_2_ under-coating corrosion layer forms, typically two to three times thinner than that observed on bare Mg. Importantly, this corrosion zone is spatially restricted, allowing the MAO coating to remain fully adherent and structurally intact throughout immersion. This localized and limited corrosion behavior explains the exceptionally low hydrogen-evolution rate (Figure 7) of the CaPSe_100 coating and its remarkably low degradation rate (~0.012 mm/year). It also correlates with the microstructural features identified for this coating (Figure 8), including its well-defined inner layer, relatively uniform topography, and controlled porosity. Although thin regions of under-coating hydration were detected in cross-sections, these zones remained continuous and mechanically stable, indicating that the coating effectively maintained its protective functionality. The schematic therefore illustrates two contrasting situations: a thick, unstable, and cracked corrosion layer forming on bare Mg versus a thin, localized corrosion zone beneath an intact MAO coating.

## 4. Conclusions

The study evaluated the effects of AC-MAO process parameters and electrolyte composition on the structure, composition, and overall performance of magnesium-based coatings. The results confirmed that the frequency of the applied current plays a critical role in shaping the final coating characteristics and functional behaviors. In general, the coatings formed were porous and structurally developed, with their thickness decreasing progressively with increasing frequency. All modifications led to a substantial reduction in water contact angle—from approximately 77° for uncoated magnesium to 8–18° for the treated specimens—suggesting enhanced surface hydrophilicity, which may promote protein adsorption and facilitate initial cell adhesion. The Ca/P ratio remained relatively consistent across all coatings, ranging from 0.08 to 0.11, indicating stable incorporation of these elements into the oxide matrix. Notably, the highest selenium content (1.30 wt.%) was observed in the specimen treated at 100 Hz, which also exhibited the greatest porosity and the most uniform surface topography, as indicated by the lowest S10z values. This coating also demonstrated a more homogeneous morphology with fewer surface defects, while coatings formed at higher frequencies showed increased heterogeneity and a tendency toward brittleness, potentially compromising long-term stability. Among all the tested coatings, the CaPSe_100 variant presented the most favorable balance of protective and functional properties. It combined high porosity, strong hydrophilicity, uniform surface features, and efficient selenium incorporation, which collectively contributed to a remarkably low degradation rate (~0.012 ± 0.003 mm/year)—well below clinically accepted thresholds. These findings demonstrate that the CaPSe_100 coating effectively suppresses early stage of magnesium degradation while providing surface characteristics that could support biological integration. This has clear practical relevance for implant design, where controlled corrosion and favorable tissue interactions are essential. While hydrogen evolution testing reflects a clinically relevant degradation behavior, future work should focus on revealing the corrosion mechanism using AC and DC electrochemical testing, ideally performed in a more complex biological media, to better understand the barrier layer behavior and coating stability. In addition, in vitro biological assays are required to confirm the cytocompatibility and potential antibacterial effects of Ca–P–Se incorporation. Despite these limitations, the results indicate that frequency-controlled AC-MAO is a promising strategy for creating multifunctional, corrosion-resistant coatings on magnesium. Such coatings are particularly relevant for biodegradable implant applications where controlled degradation and early mechanical stability are critical, including orthopedic fixation devices (e.g., screws, pins, plates), maxillofacial implants, and small load-bearing scaffolds.

## Figures and Tables

**Table 1:** Specimen designations, AC-MAO treatment durations, and suspension properties (composition, pH, conductivity (σ)) used for Mg modification.

Specimen	AC MAO Frequency (Hz)	Electrolyte					
		Concentration in Aqueous Solution (g/L)				pH	σ (mS/cm)
		NaOH	Ca(H_2_PO_4_)_2_·H_2_O	C_6_H_18_O_24_P_6_	Na_2_SeO_3_		
Mg	–	–	–	–	–	–	–
CaPSe_50	50	8	6	4	4	~13.0 ± 0.01 *	~23.9 ± 0.12 **
CaPSe_100	100						
CaPSe_200	200						
CaPSe_400	400						

* Measuring accuracy of the CRISON BASIC 20; ** Measuring accuracy of the CRISON/EC-Meter GLP 31 is 0.5%.

**Table 2:** Average pore size and porosity of the MAO coatings, as well as coating thickness (*n* = 10).

Specimen	Average Pore Size (μm^2^)	Porosity (%)	Thickness (μm)
CaPSe_50	~1.05	~24.4	8.59 ± 0.71
CaPSe_100	~1.32	~28.4	7.75 ± 0.73
CaPSe_200	~1.00	~23.3	7.57 ± 0.81
CaPSe_400	~1.08	~23.7	6.48 ± 0.66 ^&^

^&^ Statistically significant difference compared to CaPSe_50 (*p* < 0.05). No other statistically significant differences were observed between groups for coating thickness in this study.

**Table 3:** EDS-determined chemical composition (wt.%) of modified UMAO specimens from areas at 1000× magnification.

Specimen	Chemical Composition						Ratio			
	O	P	Ca	Mg	Se	Na	Ca/P	Ca/Mg	P/Mg	Se/Na
Mg	-	-	-	100	-	-	-	-	-	-
CaPSe_50	44.52	13.29	1.48	35.71	0.94	4.07	~0.11	~0.04	~0.37	~0.23
CaPSe_00	44.75	13.10	1.10	35.80	1.30	3.95	~0.08	~0.03	~0.37	~0.33
CaPSe_200	45.43	13.78	1.09	35.52	0.86	3.33	~0.08	~0.03	~0.39	~0.26
CaPSe_400	44.57	13.40	1.07	35.76	1.23	3.96	~0.08	~0.03	~0.37	~0.31

Typical EDS uncertainty: ±1–2 wt.% (>10 wt.%), ±5–10% (1–10 wt.%), ±0.2 wt.% (<1 wt.%).

## Data Availability

Data presented in this work will be made available through *Most Wiedzy* repository: https://mostwiedzy.pl/pl/open-research-data/catalog (accessed on 1 December 2025).
